# Comparison of clinical outcomes between fresh embryo transfers and frozen-thawed embryo transfers

**Published:** 2014-06

**Authors:** Chunjuan Shen, Defeng Shu, Xiaojie Zhao, Ying Gao

**Affiliations:** *Department of Obstetrics and Gynecology, Union Hospital, Tongji Medical College, Huazhong University of Science and Technology, Wuhan Hubei 430022, China. *

**Keywords:** *In vitro fertilization*, *Embryo transfer*, *Clinical outcome*, *Ectopic pregnancy*, *Endometrial receptivity*

## Abstract

**Background:** Advances in embryo culture technology and cryopreservation have led to a shift in in vitro fertilization (IVF) from early fresh or frozen-thawed cleavage embryo transfer to fresh or frozen-thawed blastocyst stage transfer.

**Objective:** To compare the clinical outcomes of fresh embryo transfers and frozen-thawed embryo transfers.

**Materials and Methods: **In this retrospective case control study, patients undergoing IVF cycles from January 2012 to December 2012 were enrolled in Assisted Reproduction of Wuhan Union Hospital were enrolled. A total of 1891 cycle contains 1150 fresh embryo transfers and 741 frozen-thawed embryo transfers were studied. All data were transferred directly to SPSS 18 and analyzed.

**Results: **Clinical pregnancy rates of fresh cleavage-stage embryo transfers compared with fresh blastocyst transfers, frozen-thawed cleavage-stage embryo transfers, post thaw cleavage-stage extended blastocyst culture transfers and frozen-thawed blastocyst transfers were 52.7%, 35.88%, 35.29%, 47.75%, 59.8% in patients under 35 years of ages and 41.24%, 26.92%, 11.32%, 46.15%, 55.8% in patients older than 35 years old, respectively. The multiple pregnancy rates, abortion rates and ectopic pregnancy rates did not differ significantly among the five groups.

**Conclusion:** The clinical pregnancy rates were not different significantly between fresh cleavage-stage embryo transfers and fresh blastocyst transfers. But the clinical pregnancy rate of frozen-thawed blastocyst transfer was the highest among fresh/frozen-thawed embryo transfers.

## Introduction

The first cleavage-stage embryo was transferred by Edwards in 1978. After five years, Trounson transferred the first frozen-thawed embryo and the patient get pregnant (1). With the development of assisted reproductive technology (ART), fresh blastocysts transfer, frozen-thawed cleavage-stage embryo transfer, and frozen-thawed blastocyst transfer had good pregnancy outcomes. However, how to promote pregnancy rate is the focus of attention. Embryo and endometrial receptivity are the important factors. Blastocyst culture can select available embryo through extended culture, on the other hands, the better synchronization of embryo-endometrium improve endometrial receptivity (2). 

Recently, many researches showed frozen-thawed embryo transfers can enhanced the embryo utilization rate and improve the success rate (3, 4). Estrogen-progesterone (E-P) cycles or nature cycles were adopted in frozen-thawed embryo transfer (3). This study was aimed to compare the fresh embryo transfer with frozen-thawed embryo transfer to evaluate an optimal embryo transfer protocol. 

## Materials and methods

The study performed from Jan 2012 to Dec 2012 in Assisted Reproduction of Wuhan Union Hospital. A total of 1891 cycle contains 1150 fresh embryo transfers and 741 frozen-thawed embryo transfers. The 1150 women were composed with cleavage-stage (n=799 <35 years old and n=194 ≥35 years old, respectively) or blastocyst-stage (n=131, <35 years old and n=26, ≥35 years old, respectively). 741 women also were divided into cleavage-stage (n=159, <35 years old and n=53, ≥35 years old, respectively) or cleavage-stage extended blastocyst culture (n=111, <35 years old and n=26, ≥35 years old respectively) or blastocyst-stage transfer (n=276, <35 years old and n=52, ≥35 years old, respectively). 

**Process of IVF**

A long gonadotropin releasing hormone (GnRH) agonist protocol was used in all cycles. When two or more follicles reached 18 mm in diameter, 10000 units of HCG was injected and then ovum pick up (OPU) was performed 34-36 hours later. In vitro fertilization (IVF) or Intracytoplasmic sperm injection (ICSI) was performed 4 to 6 hours after OPU. Embryo assessment was based on morphology and rate of development. The grade of D3 embryos were scored on a scale of 1-4, where 1 and 2 were good embryo, 3-4 were bad embryo. Then we performed D3 embryo transfer or transferred them to blastocyst medium, culturing for further 2-3 days to form blastocyst. Blastocysts were scored according to Gardner standard (5). 

We usually transferred 1 or 2 good embryos. All surplus good quality embryos were cryopreserved through vitrification. The E-P cycle or nature cycle was administrated according to the characteristics of patients. The double thickness of the endometrium which exceeded 8 mm was available. Clinical pregnancy was defined by the observation of a gestational sac with or without a fetal heartbeat on ultrasound evaluation on the 30^th^ day after embryo transfer. The number of sacs was taken as the number of implantations.

**Statistical analysis**

All statistical calculations were done by SPSS software. Logistic regression analysis was used to adjust the confounding factors to compare the embryo clinical pregnancy rates between the two groups. P<0.05 was considered statistically significant.

## Discussion

For patients under 35 years old, a total of 930 fresh embryo transfers were assigned to fresh cleavage-stage embryo (n=799) and fresh blastocyst transfers (n=131). There are significant differences in some clinical characteristics (age, the number of oocyte and the number of transferring good-quality embryo) between two groups (Table I). The clinical pregnancy rates in the fresh cleavage-stage embryo and fresh blastocyst transfers were 52.7% and 35.88% (p<0.0001). But the multiple pregnancy rates had no statistically significant difference between the two groups (34.92% vs. 29.79%), also the same were the abortion rates and ectopic pregnancy rates (5.7% vs. 4.25%, and 3.32% vs. 0%). For patients older than 35 years old, fresh cleavage-stage embryo (n=194) and fresh blastocyst transfer (n=26) have no difference in clinical characteristics (Table II). There are significantly different in clinical pregnancy rates between two groups (41.24% vs. 26.92%). There were no significant differences between the two groups with respect to multiple pregnancy rates, abortion rates and ectopic pregnancy rates.

A total of 741 frozen-thawed embryo transfers were assigned to frozen-thawed cleavage-stage embryo transfers (n=159, <35 years and n=53, ≥35 years), post-thaw extended culture blastocyst transfers (n=111, <35 years and n=26, ≥35 years) and frozen-thawed blastocyst transfers (n=276, <35 years and n=52, ≥35 years) (Table III and IV). The clinical pregnancy rates of the frozen-thawed cleavage-stage embryo transfers and frozen-thawed blastocyst transfers were 35.29% and 59.8% (<35 years old), and 11.32% and 55.8% (≥35 years old) (p<0.0001). Frozen-thawed blastocyst transfers versus post thaw extended culture blastocyst transfers were 47.75% vs. 59.8% (<35 years old), and 46.15% vs. 55.8% (≥35years old) (p<0.0001). 

The multiple pregnancy rates after frozen-thawed cleavage-stage embryo transfers, post-thaw extended culture blastocyst transfers and frozen-thawed blastocyst transfer were 31.48%, 33.96% and 31.52% (<35 years), and 16.67%, 25% and 17.24% (≥35 years). Abortion rates after frozen-thawed cleavage-stage embryo transfers, post-thaw extended culture blastocyst transfers and frozen-thawed blastocyst transfers were 9.26%, 13.21% and 10.9% (<35 years), and 33.33%, 16.67% and 17.24% (≥35 years). 

Ectopic pregnancy rates after frozen-thawed cleavage-stage embryo transfers, post-thaw extended culture blastocyst transfers and frozen-thawed blastocyst transfers were 0, 3.78% and 1.82% (<35 years), and 0%, 0% and 3.45% (≥35 years). There were no significantly different among three group with respect to clinical outcomes except for clinical pregnancy rate whatever the patients under 35 years or older than 35 years. 

There were no differences between the fresh cleavage-stage embryo transfers and frozen-thawed cleavage-stage embryo transfers in the clinical characteristics of age, and dosage of gonadotropin were different in ≥35 years (Table V). The clinical pregnancy rates differ significantly between the fresh cleavage-stage embryo transfers and frozen-thawed cleavage-stage embryo transfers (52.7% vs. 35.29%, <35 years and 41.24% vs. 11.32%, ≥35 years). The multiple pregnancy rates of the fresh cleavage-stage embryo transfers and frozen-thawed cleavage-stage embryo transfers were 34.92% vs. 31.48% (<35 years) and 22.5% vs. 16.67% (≥35 years), while the abortion rates were 5.7% vs. 9.26% (<35 years) and 8.75% vs. 33.33% (≥35 years), the ectopic pregnancy rates were 3.32% vs. 0% (<35 years) and 0% vs. 0% (≥35 years). There were no different between the groups. 

For patients under 35 years of age, the numbers of good embryo transferred were different in the fresh cleavage-stage embryo transfers and frozen-thawed blastocyst transfers. The clinical pregnancy rates after the fresh cleavage-stage embryo transfers and frozen-thawed blastocyst transfers were different (52.7% vs. 59.8%). But other clinical outcomes, for example multiple pregnancy rates, abortion rates, and ectopic pregnancy rates had no difference. For patients older than 35 years old, the clinical characteristics, for example, dose of gonadotropin, retrieved of oocyte, and number of good embryo transferred were different significantly between the fresh cleavage-stage embryo transfers and frozen-thawed blastocyst transfers (41.24% vs. 55.8%). Whereas the multiple pregnancy rates, abortion rates, and ectopic pregnancy rates had no difference (Table VI).

**Table I:** comparison with fresh cleavage-stage embryo transfer and fresh blastocyst transfer (age <35 years)

	**Fresh cleavage-stage** **embryo transfer (n=799)**	**Fresh blastocyst** **transfer (n=131)**	**p-value**
Age	28.52 ± 3.21	29.16 ± 3.31	0.038
Body mass index	19.34 ± 6.22	19.04 ± 5.63	0.757
Dose of gonadotropin	29.59 ± 9.76	29.12 ± 8.53	0.925
Retrieved of oocyte	14.31 ± 6.74	17.87 ± 9.55	<0.0001
Endotrium Thickness	10.74 ± 2.33	10.71 ± 2.20	0.791
No of embryo transferred	1.98 ± 0.22	1.78 ± 0.46	<0.0001
No of good embryo transferred	1.78 ± 0.28	1.47 ± 0.41	<0.0001
Clinical pregnancy rate	52.7% (421/799)	35.88% (47/131)	<0.0001
Multiple pregnancy rate	34.92% (147/421)	29.79% (14/47)	0.266
Abortion rate	5.7% (24/421)	4.25% (2/47)	0.504
Ectopic pregnancy rate	3.32% (14/421)	0	0.222

Note: Values are presented as number, number (%) or mean±SD.

**Table II:** Comparison with fresh cleavage-stage embryo transfer and fresh blastocyst transfer (age ≥35 years)

	**Fresh cleavage-stage** **embryo transfer (n=194)**	**Fresh blastocyst** **transfer (n=26)**	**p-value**
Age	37.45 ± 2.49	38.19 ± 2.77	0.159
Body mass index	21.15 ± 6.62	21.80 ± 6.72	0.500
Dose of gonadotropin	43.20 ± 15.54	40.51 ± 13.26	0.398
Retrieved of oocyte	10.11 ± 6.41	10.88 ± 6.01	0.355
Endotrium thickness	10.49 ± 2.40	10.58 ± 2.35	0.779
No of embryo transferred	2.04 ± 0.49	2.00 ± 0.40	0.667
No of good embryo transferred	1.84 ± 0.45	1.70 ± 0.39	0.692
Clinical pregnancy rate	41.24% (80/194)	26.92% (7/26)	0.116
Multiple pregnancy rate	22.5% (18/80)	0	0.185
Abortion rate	8.75% (7/80)	14.29% (1/7)	0504
Ectopic pregnancy rate	0	0	——

Note: Values are presented as number, number (%) or mean±SD

**Table III:** Comparison with three types of frozen-thawed embryo transfers (age <35 years)

	**Frozen-thawed cleavage-stage** **embryo transfer (n=159)**	**Post-thawed extended culture** **blastocyst transfer (n=111)**	**p-value**	**Frozen-thawed** **blastocyst transfer (n=276)**
Age	28.95 ± 2.92	28.99 ± 3.16	0.768	28.59 ± 3.15
Body mass index	19.78 ± 6.45	19.35 ± 5.98	0.571	19.26 ± 6.02
Dose of gonadotropin	29.62 ± 9.07	29.47 ± 8.67	0.279	30.02 ± 8.90
Retrieved of oocyte	15.37 ± 7.02	16.45 ± 9.05	0.460	15.37 ± 8.89
Endotrium thickness	9.27 ± 1.94	9.44 ± 1.67	0.546	9.37 ± 1.69
No of embryo transferred	2.19 ± 0.48	1.85 ± 0.36	0.117	1.84 ± 0.36
No of good embryo transferred	1.82 ± 0.32	1.46 ± 0.31	0.047	1.45 ± 0.28
Clinical pregnancy rate	35.29% (54/153)	47.75% (53/137)	<0.0001	59.8% (165/276)
Multiple pregnancy rate	31.48% (17/54)	33.96% (18/53)	0.943	31.52% (52/165)
Abortion rate	9.26% (5/54)	13.21% (7/53)	0.627	15.38% (8/52)
Ectopic pregnancy rate	0	3.78% (2/53)	0.223	1.82% (3/52)

Note: Values are presented as number, number (%) or mean±SD

**Table IV:** Comparison with three types of frozen-thawed embryo transfers (age >35 years)

	**Frozen-thawed cleavage-stage** **embryo transfer (n=53)**	**Post-thawed extended culture** **blastocyst transfer (n=26)**	**p-value**	**Frozen-thawed** **blastocyst transfer (n=52)**
Age	39.13 ± 3.46	37.76 ± 3.23	0.047	37.57 ± 2.52
Body mass index	21.56 ± 6.73	22.69 ± 6.97	0.321	22.72 ± 7.09
Dose of gonadotropin	39.28 ± 15.78	38.90 ± 14.57	0.794	39.0 ± 15.80
Retrieved of oocyte	11.46 ± 6.98	12.09 ± 6.78	0.237	12.98 ± 7.04
Endotrium thickness	8.97 ± 1.74	9.17 ± 1.96	0.643	9.63 ± 1.83
No of embryo transferred	2.30 ± 0.61	1.77 ± 0.43	0.033	1.71 ± 0.46
No of good embryo transferred	2.01 ± 0.42	1.56 ± 0.27	0.027	1.49 ± 0.30
Cryosurvival	93.84%	94.45%	0.306	95.09%
Clinical pregnancy rate	11.32% (6/53)	46.15% (12/26)	<0.0001	55.8% (29/52)
Multiple pregnancy rate	16.67% (1/6)	31.52% (52/165)	0.235	17.24% (5/29)
Abortion rate	33.33% (2/6)	10.9% (18/52)	0.245	17.24% (5/29)
Ectopic pregnancy rate	0	1.82% (3/52)	0.764	3.45% (1/29)

Note: Values are presented as number, number (%) or mean±SD.

**Table V:** Comparison with fresh cleavage-stage embryo transfers and frozen-thawed cleavage-stage embryo transfers

	**<35 years**		**≥35 years**	
	**Fresh cleavage-stage** **embryo transfer (n=799)**	**Frozen-thawed cleavage-stage** **embryo transfer (n=159)**	**Fresh cleavage-stage** **embryo transfer (n=194)**	**Frozen-thawed cleavage-stage** **embryo transfer (n=53 )**
Age	28.52 ± 3.21	28.95 ± 2.92	37.45 ± 2.49*	39.13 ± 3.46*
Body mass index	19.34 ± 6.22	19.78 ± 6.45	21.15 ± 6.62	21.56 ± 6.73
Dose of gonadotropin	29.59 ± 9.76	29.62 ± 9.07	43.20 ± 15.54*	39.28 ± 15.78*
Retrieved of oocyte	14.31 ± 6.74	15.37 ± 7.02	10.11 ± 6.41	11.46 ± 6.98
Endotrium thickness	10.74 ± 2.33	9.27 ± 1.94	10.49 ± 2.40	8.97 ± 1.74
No of embryo transferred	1.98 ± 0.22	2.19 ± 0.48	2.04 ± 0.49	2.30 ± 0.61
No of good embryo transferred	1.78 ± 0.28	1.82 ± 0.32	1.84 ± 0.45	2.01 ± 0.42
Clinical pregnancy rate	52.7% (421/799)*	35.29% (54/153)*	41.24% (80/194)*	11.32% (6/53)*
Multiple pregnancy rate	34.92% (147/421)	31.48% (17/54)	22.5% (18/80)	16.67% (1/6)
Abortion rate	5.7% (24/421)	9.26% (5/54)	8.75% (7/80)	33.33% (2/6)
Ectopic pregnancy rate	3.32% (14/421)	0	0	0

Note: Values are presented as number, number (%) or mean ± SD.

* was considered significant difference between fresh cleavage-stage embryo transfer and frozen-thawed cleavage-stage embryo transfer groups

**Table VI:** Comparison with fresh cleavage-stage embryo transfers and frozen-thawed blastocyst transfers

	**<35 years**		**≥35 years**	
	**Fresh cleavage-stage** **embryo transfer (n=799)**	**Frozen-thawed** **blastocyst transfer (n=276)**	**Fresh cleavage-stage** **embryo transfer (n=194)**	**Frozen-thawed bastocyst transfer (n =52)**
Age	28.52 ± 3.21	28.59 ± 3.15	37.45 ± 2.49	37.57 ± 2.52
Body mass index	19.34 ± 6.22	19.26 ± 6.02	21.15 ± 6.62	22.72 ± 7.09
Dose of gonadotropin	29.59 ± 9.76	30.02 ± 8.90	43.20 ± 15.54*	39.0 ± 15.80*
Retrieved of oocyte	14.31 ± 6.74	15.37 ± 8.89	10.11 ± 6.41*	12.98 ± 7.04*
Endotrium thickness	10.74 ± 2.33	9.37 ± 1.69	10.49 ± 2.40	9.63 ± 1.83
No of embryo transferred	1.98 ± 0.22	1.84 ± 0.36	2.04 ± 0.49	1.71 ± 0.46
No of good embryo transferred	1.78 ± 0.28*	1.45 ± 0.28*	1.84 ± 0.45*	1.49 ± 0.30*
Clinical pregnancy rate	52.7% (421/799)*	59.8% (165/276)*	41.24% (80/194)*	55.8% (29/52)*
Multiple pregnancy rate	34.92% (147/421)	31.52% (52/165)	22.5% (18/80)	17.24% (5/29)
Abortion rate	5.7% (24/421)	10.9% (18/52)	8.75% (7/80)	17.24% (5/29)
Ectopic pregnancy rate	3.32% (14/421)	1.82% (3/52)	0	3.45% (1/29)

Note: Values are presented as number, number (%) or mean ± SD.

* was considered significant difference between fresh cleavage-stage embryo transfer and frozen-thawed blastocyst transfer groups

## Discussion

The study showed a higher clinical pregnancy rate in fresh cleavage-stage embryo transfers than frozen-thawed cleavage-stage transfers. But some researches claimed fresh embryo transfers had a lower clinical pregnancy rate than frozen-thawed embryo transfers (3, 6). There was a difference that the embryos were included cleavage-stage and blastocyst. Our research also showed the clinical pregnancy rates were significantly highest in the frozen-thawed blastocyst transfer among fresh embryo transfers and frozen-thawed embryo transfers. We inferred that the ability of development in frozen-thawed cleavage-stage embryos may be lower than the fresh cleavage-stage embryos. 

The influence of blastocyst after cryopreservation may be little. The cleavage-stage embryos did not induce self-selection through the extended culture. So the ability of resistance may be weaker than blastocyst. The result was in according with many studies (3, 7, 8). One study indicated that high serum oestradiol concentrations may adversely affect endometrial receptivity, but the embryo quality was not affected (9). Morphology study of Basir *et al* revealed that high oestradiol concentrations in COH cycles induced delayed glandular maturation and advanced stromal morphology, whereas moderate oestradiol concentrations showed synchronous development of grandular and stromal features. Endometrial characteristic of pre-ovulation were different between the COH cycles and natural cycles (10, 11). Gene study of Horcajadas *et al* demonstrated more than 200 genes had abnormal expression during the window of implantation when COH cycle compared with normal cycles. Gene expression profiles of the endometrium during COH produced a suboptimal endometrial development (12).

Liu *et al* found there were lots of abnormal morphology oocytes and delayed development and maturation in high dose of oestradiol. Finally the oocytes would be differently fertilized by sperms                    (13). The oestradiol concentrations in COH cycles were inevitably higher than this in the nature cycles. We also found the clinical pregnancy rates of fresh blastocyst transfers were lower than fresh cleavage-stage embryo transfers. Some studies showed the opposite outcomes (14). But some other reports support us (8, 15). In our centre, we usually transfer fresh cleavage-stage embryo in fresh cycles. Yet when the patients have some clinical symptoms, for example OHSS, pelvic effusion, endometrial cavity fluid, thick endometrium, a failure or more after IVF-ET, we cultured the embryos 2-3 days until blastocyst formed, then transferred the blastocyst if the patients were in good condition. This condition may influence pregnancy outcomes. 

Recently, studies indicated there were high ectopic pregnancy rates in fresh embryo transfer cycles. The reason may be the high gonadotropin in ovarian stimulation cycles (16). The statistics in our study showed no difference in the ectopic pregnancy between fresh embryo transfers and frozen-thawed embryo transfers. Maybe, fresh and frozen-thawed embryo transfers were divided into cleavage-stage and blastocyst to analyze. The abortion rate in frozen-thawed embryo transfer was higher than fresh embryo transfer. Some reports supported the view (17, 18).

Cleavage-stage embryo and blastocyst may inevitably be damaged during cryopreservation. This may damage the ability of development, thus result in abortion (18). Aytoz *et al* indicated the cryopreservation process had no negative impact on the outcome of pregnancy over 20 weeks of gestation (17). There were no differences in low-birthweight, birth defects and perinatal death between the fresh embryo transfers and frozen-thawed embryo transfers (19).

## Conclusion

In conclusion, the optimal embryo transfer protocol is fresh cleavage-stage embryo transfer if the patients are in good conditions. However, if the patients have some complications such as high oestradiol concentrations on, OHSS, the optimal embryo transfer protocol is culture cleavage-stage embryo to blastocyst and cryopreservation. The clinical pregnancy rates in frozen-thawed blastocyst transfers were high.
